# Cath-Lab Clues to Type A Aortic Dissection Presenting as Acute Myocardial Infarction

**DOI:** 10.1016/j.jaccas.2026.109923

**Published:** 2026-08-22

**Authors:** Yingdong Wang, Yuhe Sheng, Yaling Han, Xiaozeng Wang, Bin Wang

**Affiliations:** a Department of Cardiology, General Hospital of Northern Theater Command, Shenyang, Liaoning, China; b Lanzhou University, Lanzhou, Gansu, China

**Keywords:** acute myocardial infarction, acute type A aortic dissection, coronary angiography, coronary malperfusion, percutaneous coronary intervention

## Abstract

**Image(s):**

Emergency coronary angiography and computed tomography angiography showing catheterization laboratory (Cath-Lab) clues to type A aortic dissection presenting as acute myocardial infarction.

**Case Summary:**

A 67-year-old man underwent emergency angiography for anterior ST-segment elevation. Difficult coronary engagement, thin strip-like left coronary narrowing, and marked catheter swing prompted reassessment. Computed tomography angiography confirmed type A aortic dissection involving the left main ostium, and unnecessary percutaneous coronary intervention was avoided. Five recurring angiographic warning clues are summarized.

**Take-Home Messages:**

These 5 practical Cath-Lab clues would help you away from emergency type A aortic dissection trap. Timely recognition of Cath-Lab clues and pause; activate the aortic surgical team.

## Visual Summary

**Figure:**
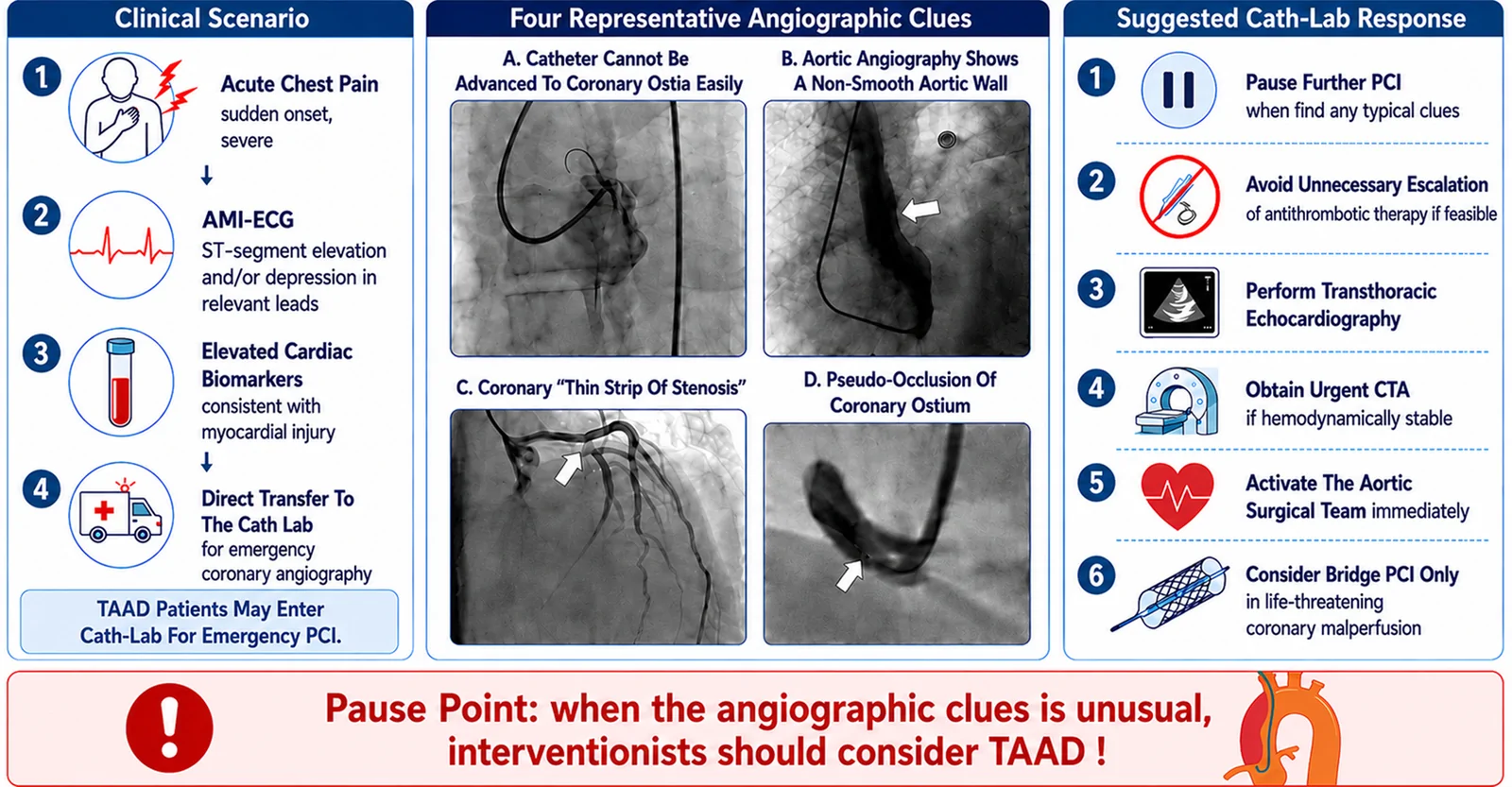

A 67-year-old man presented with acute chest pain and was initially treated for suspected acute myocardial infarction (AMI). Electrocardiography showed extensive ST-segment elevation in the anterior leads and ST-segment depression in the inferior leads (Figure 1A1). He was transferred to the catheterization laboratory (Cath-Lab) for emergency coronary angiography.

**Figure 1:**
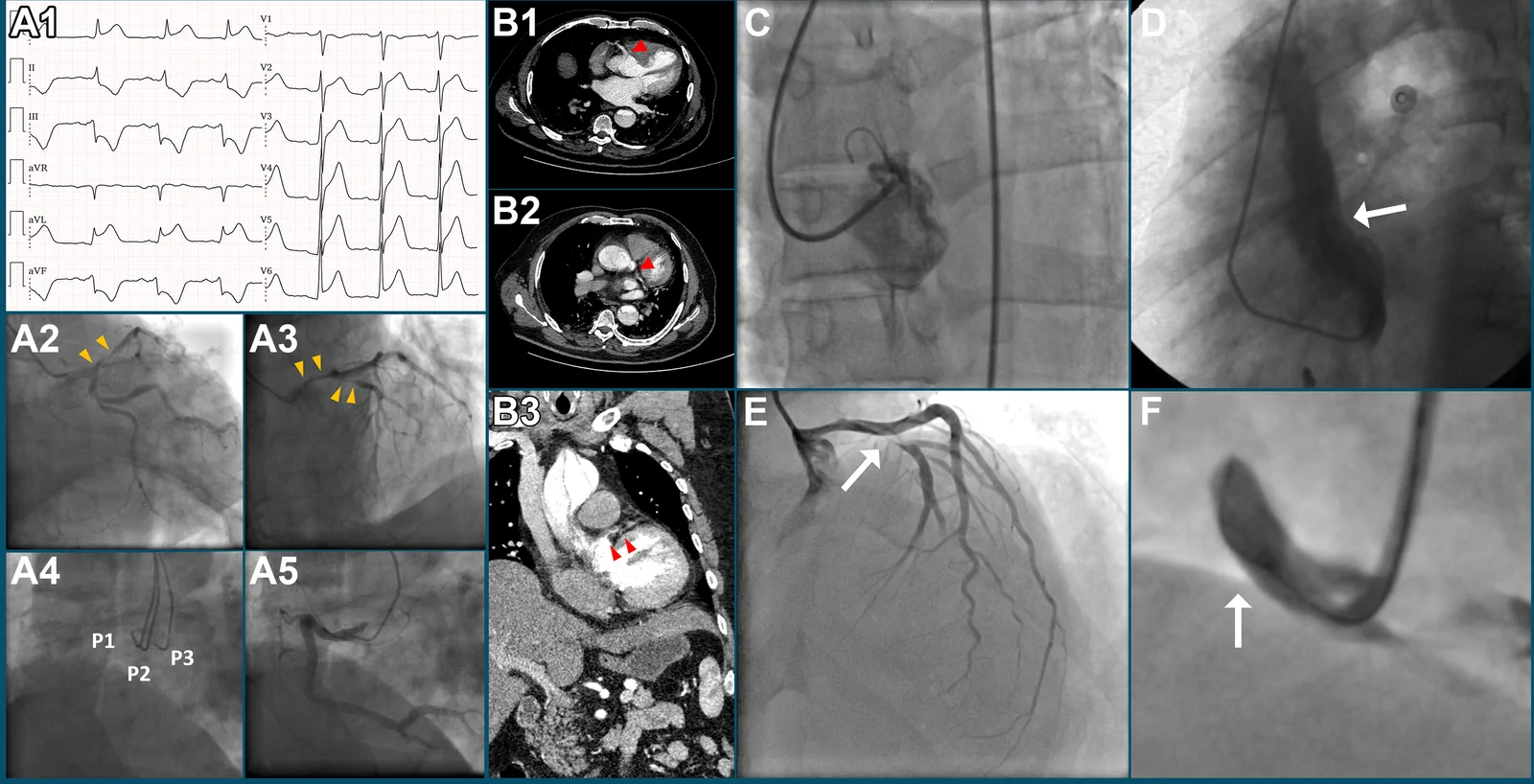
Electrocardiography, Angiography and Emergency Computed Tomography Images for the Patient and Catheterization-Laloratory Clues (A1) Electrocardiography showed extensive ST-segment elevation in the anterior leads and ST-segment depression in the inferior leads. (A2 and A3). Severe stenosis of the left main and proximal left anterior descending artery. (A4) Significant catheter swing in the aorta; P1, P2, and P3 showed different positions when the catheter was swinging. (A5) The right coronary artery was normal. (B1) The right coronary artery originated from the true lumen. (B2 and B3) The intimal flap involved the left main coronary ostium. (C) The catheter cannot be advanced to the coronary ostia easily. (D) Aortic angiography shows a nonsmooth aortic wall (white arrow). (E) The coronary “thin strip of stenosis” (white arrow). (F) Pseudo-occlusion of the coronary ostium (white arrow). aVF = augmented vector foot; aVL = augmented vector left; aVR = augmented vector right; V_1_-V_6_ = precordial leads V_1_ through V_6_.

Selective coronary engagement was difficult. Angiography showed severe stenosis of the left main and proximal left anterior descending artery (Figures 1A2 and 1A3, Video 1). However, the stenosis appeared atypical, presenting as a coronary “thin strip of stenosis” rather than a typical atherosclerotic culprit lesion. Significant catheter swing in the aorta was also observed when the catheter was turned toward the right coronary ostium (Figure 1A4, Video 1), whereas the right coronary artery was angiographically normal (Figure 1A5). These findings raised suspicion for type A aortic dissection (TAAD), and balloon angioplasty or stent implantation was not performed. Emergency computed tomography aortography confirmed TAAD, showing that the right coronary artery originated from the true lumen and that the intimal flap involved the left main coronary ostium, explaining the abnormal catheter behavior and the stenosis of the left coronary artery (Figures 1B1 to 1B3). The patient underwent surgical treatment and survived.

This index case prompted a retrospective review of 19 patients with confirmed TAAD who initially presented with suspected AMI and underwent emergency coronary angiography at our institution between January 2008 and November 2022. Angiograms, aortograms, procedural records, and final imaging diagnoses were reviewed. Five recurring catheterization-laboratory clues were identified: catheters could not be advanced to the coronary ostia easily (Figure 1C, Video 2); significant catheter swing in the aorta (Video 3); aortic angiography showed a nonsmooth aortic wall (Figure 1D); coronary “thin strip of stenosis” (Figure 1E); and pseudo-occlusion of the coronary ostium (Figure 1F). At least 1 clue was present in 14 of 19 patients (73.7%). Detailed definitions of these clues and their potential mechanisms are provided in the Supplemental Appendix because of space limitations.

To assess whether similar findings had been reported, we screened English-language publications in PubMed. Twenty-eight published cases of TAAD presenting as AMI and undergoing coronary angiography were identified. Similar angiographic findings were described, with coronary “thin strip of stenosis” being the most frequent clue (details are provided in the Supplemental Appendix).

TAAD is associated with high mortality^1^ and may be difficult to recognize when AMI is the initial presentation. AMI guidelines emphasize timely reperfusion, whereas suspected TAAD requires prompt echocardiography or computed tomography for confirmation.^2^ These Cath-Lab clues are intended to serve not as reasons to delay reperfusion in typical AMI, but as a practical “pause point” when angiographic findings are inconsistent with routine culprit-lesion disease. The pause should trigger reassessment of the working diagnosis, rapid aortic evaluation, and surgical or multidisciplinary aortic consultation. In selected patients with life-threatening dissection-related coronary malperfusion, percutaneous coronary intervention may be considered as bridge therapy while definitive aortic repair is being arranged.^3^

**Visual Summary:**
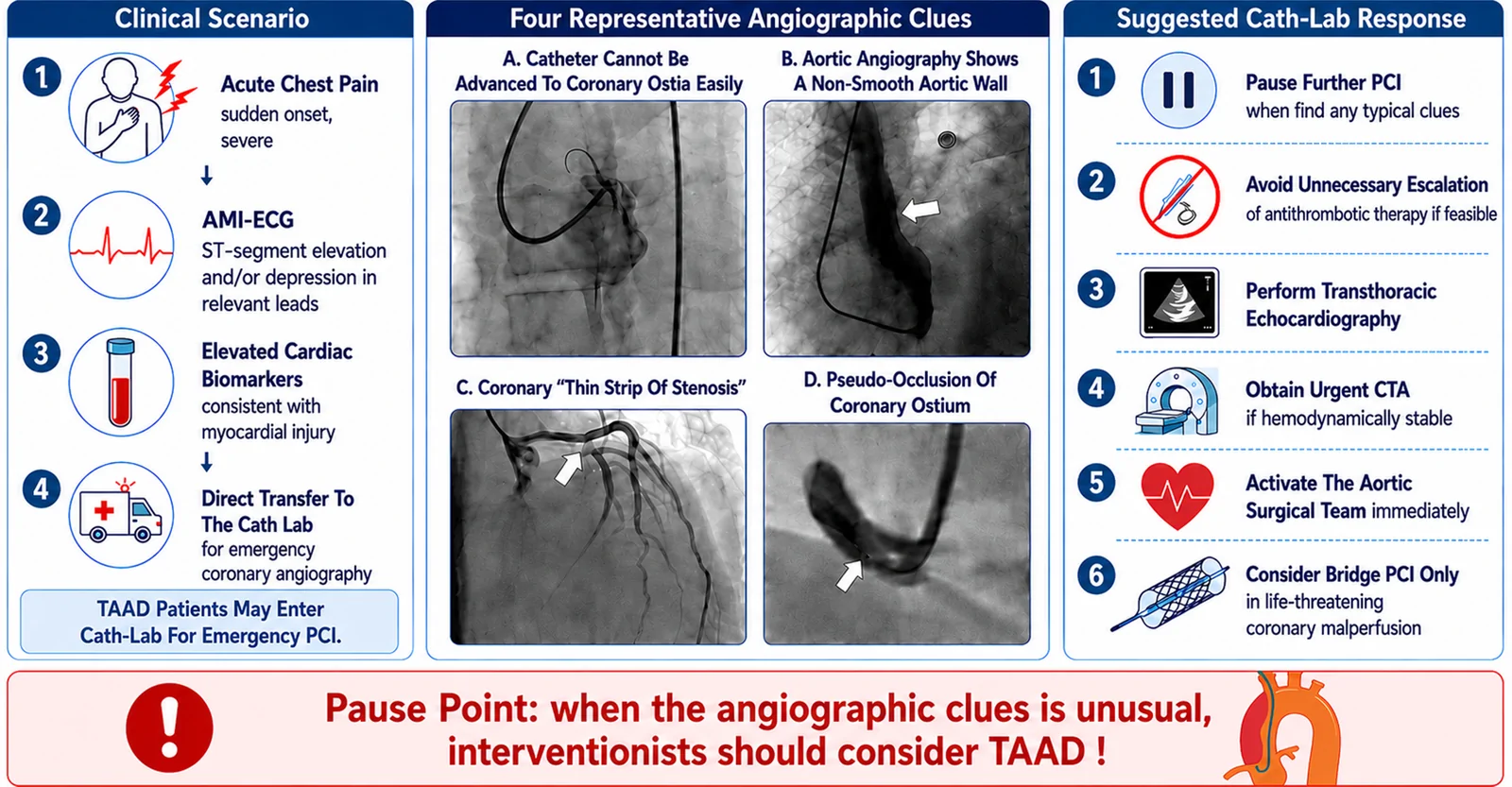
Cath-Lab Clues to Type A Aortic Dissection Presenting as Acute Myocardial Infarction: From Misdiagnosis to Recovery Panel Clinical Scenario: Patients with type A aortic dissection can likely be transferred to the Cath-Lab for angiography. Panel Cath-Lab Clues: A. Catheters cannot be advanced to the coronary ostia easily; B. Aortic angiography shows a non-smooth aortic wall; C. Coronary "thin strip of stenosis"; D. Pseudo-occlusion of the coronary ostium; ‘Significant catheter swing within the aorta’ was showed in Video 3. Panel Suggested Cath-Lab response: What should be done after identifying the Cath-Lab clues. Cath-Lab = catheterization laboratory; CTA = computed tomography angiography; ECG = electrocardiogram; PCI = percutaneous coronary intervention; TAAD = type A aortic dissection.

## Funding Support and Author Disclosures

This work was supported by the Liaoning Provincial Science and Technology Joint Project (grant number 2025JH2/101800031) and the National Key R&D Program of China (grant number 2020YFC2008100). The authors have reported that they have no relationships relevant to the contents of this paper to disclose.

**Take-Home Messages**

• These 5 practical Cath-Lab clues would help you away from emergency type A aortic dissection trap.
• Timely recognition of Cath-Lab clues and pause; activate the aortic surgical team.

## Appendix

Video 1 Angiographic Images of Index Patient: Three Cath-Lab Clues Included: Catheters Could not be Advanced to the Coronary Ostia Easily; Significant Catheter Swing in the Aorta; Coronary “Thin Strip of Stenosis

Video 2 Catheters Cannot be Advanced to the Coronary Ostia Easily

Video 3 Significant Catheter Swing Within the Aorta
