# Supplementary material for: Cath-Lab Clues to Type A Aortic Dissection Presenting as Acute Myocardial Infarction
Source: JACC Case Rep. 2026 Aug 22;31(42):109923. doi: 10.1016/j.jaccas.2026.109923 (PMC13698984; doi:10.1016/j.jaccas.2026.109923)
Supplement: Supplementary file 1 [file mmc4.docx]

## Supplementary Materials

## 1. Table S1. Baselines, clinical characteristics and outcomes of institutional cases

| Variables | Patients |
| --- | --- |
|  | n=19 |
| Age, mean (SD), y | 50.05±9.17 |
| Male, NO. (%) | 16(84.2%) |
| Type of MI, NO. (%) |  |
| STEMI | 10(52.6%) |
| NSTEMI | 9(47.4%) |
| Medical history, NO. (%) |  |
| Hypertension | 12(63.2%) |
| Smoking | 10(52.6%) |
| History of coronary artery disease | 1(5.3%) |
| Emergency coronary angiography | 19(100%) |
| Underwent coronary-intervention | 6(31.6%) |
| Method of diagnosis |  |
| Aortography | 10(52.6%) |
| CT | 6(31.6%) |
| Post-analysis | 3(15.8%) |
| Underwent surgery, NO. (%) | 12(63.2%) |
| 30-day mortality after surgery, NO. (%) | 4/12(33.3%) |
| Total 30-day mortality, NO. (%) | 11(57.9%) |

**Abbreviations:** MI, myocardial infarction; STEMI, ST-segment elevated myocardial infarction; NSTEMI, non-ST-segment elevated myocardial infarction; CT, computed tomography.

**2. Table S2. Institutional patients’ numbers and angiographic clues.**

| **Patients-Number** | **Age** | **Gender** | **STEMI** | **Catheters cannot be advanced to the coronary ostia easily** | **Significant catheter Swing within the aorta** | **Aortic angiography shows a non-smooth aortic wall** | **Coronary thin strip of stenosis** | **Pseudo-occlusion of the coronary ostium** |
| --- | --- | --- | --- | --- | --- | --- | --- | --- |
| 1 | 46 | male | yes | yes | no | yes | no | yes |
| 2 | 34 | male | no | no | no | no | no | no |
| 3 | 45 | male | no | no | no | no | no | no |
| 4 | 47 | male | yes | yes | yes | no | no | no |
| 5 | 57 | female | yes | no | no | no | yes | no |
| 6 | 47 | male | yes | no | yes | yes | yes | yes |
| 7 | 48 | male | yes | yes | yes | no | no | no |
| 8 | 59 | female | no | no | no | no | no | no |
| 9 | 46 | male | yes | yes | yes | yes | no | no |
| 10 | 41 | male | no | yes | no | yes | no | no |
| 11 | 31 | male | yes | yes | no | yes | no | yes |
| 12 | 54 | male | yes | yes | no | yes | no | no |
| 13 | 52 | male | no | no | no | no | no | no |
| 14 | 66 | female | no | yes | yes | yes | no | no |
| 15 | 52 | male | no | no | no | no | no | no |
| 16 | 57 | male | no | no | yes | no | no | no |
| 17 | 52 | male | yes | yes | yes | no | no | no |
| 18 | 42 | male | no | yes | yes | yes | no | no |
| 19 | 67 | male | yes | yes | yes | no | yes | no |

**Abbreviations:** STEMI, ST-segment elevated myocardial infarction.

**3.The flow chart of literatures search**

The search terms included “aortic dissection,” “type A aortic dissection,” “ST elevation myocardial infarction,” “percutaneous coronary intervention,” “aortography,” and “case reports.” These terms were used in various combinations to find relevant articles. After deduplication, the search results were independently reviewed by two reviewers based on titles and abstracts. Following the search and identification of eligible studies, the reference lists of the included articles were manually searched for additional relevant studies (the flow chart is included)

**4. Table S3. Data from the published literatures.**

| **Patient Number** | **Age** | **Gender** | **Type of AMI** | **Involved**  **coronary**  **artery** | **Cath-lab clues or not** | **Type of**  **clues** | **Stents were implanted or not** | **Method of diagnosis** | **Clinical outcome** | **Involved**  **media** | **Reference** |
| --- | --- | --- | --- | --- | --- | --- | --- | --- | --- | --- | --- |
| 1 | 48 | male | STEMI | RCA | yes | **④** | yes | CT | alive | image | 1 |
| 2 | 42 | male | STEMI | RCA | yes | **④** | yes | CT | alive | image | 2 |
| 3 | 54 | male | STEMI | LCA | yes | **④** | yes | ANG | dead | image | 3 |
| 4 | 66 | female | STEMI | LCA | yes | **④** | yes | CT | dead | image | 4 |
| 5 | 57 | female | STEMI | LCA/RCA | yes | **④** | yes | CT | alive | image | 5 |
| 6 | 57 | male | STEMI | LCA/RCA | yes | **①.②.④** | yes | CT | alive | video | 6 |
| 7 | 51 | male | STEMI | LCA | yes | **④** | yes | ECHO | alive | image | 7 |
| 8 | 58 | male | STEMI | Unknown | yes | **①** | no | CT | dead | image and video | 8 |
| 9 | 49 | male | STEMI | LCA | yes | **④** | yes | ECHO and CT | alive | image | 9 |
| 10 | 81 | female | STEMI | RCA | yes | **②.④** | yes | CT | alive | image | 10 |
| 11 | 48 | male | STEMI | LCA | yes | **④** | yes | ECHO | dead | image | 11 |
| 12 | 67 | male | STEMI | LCA/RCA | yes | **①.②.** | no | ANG and ECHO | alive | image and video | 12 |
| 13 | 72 | male | STEMI | LCA | yes | **④** | yes | IVUS and CT | unknown | image and video | 13 |

| **Patient Number** | **Age** | **Gender** | **Type of AMI** | **Involved**  **coronary**  **artery** | **Cath-lab clues or not** | **Type of**  **clues** | **Stents were implanted or not** | **Method of diagnosis** | **Clinical outcome** | **Involved**  **media** | **Reference** | |
| --- | --- | --- | --- | --- | --- | --- | --- | --- | --- | --- | --- | --- |
| 14 | 80 | male | STEMI | RCA | yes | **①.②.** | no | ANG | unknown | image and video | | 14 |

| 15 | 66 | male | STEMI | RCA | yes | **④** | yes | CT | alive | image | 15 |
| --- | --- | --- | --- | --- | --- | --- | --- | --- | --- | --- | --- |
| 16 | 70 | male | STEMI | RCA | yes | **④** | yes | CT | dead | image | 16 |
| 17 | 51 | male | STEMI | LCA | yes | **④** | yes | IVUS and CT | alive | image | 17 |
| 18 | 70 | male | STEMI | LCA | yes | **④** | yes | ANG | alive | image | 18 |
| 19 | 52 | male | STEMI | LCA | yes | **④** | yes | IVUS | dead | image | 19 |
| 20 | 73 | female | NSTEMI | RCA/LCA | yes | **①.⑤.** | no | ANG | alive | image and video | 20 |
| 21 | 62 | male | STEMI | LCA | yes | **①** | no | ANG | alive | image | 21 |
| 22 | 56 | male | STEMI | LCA | yes | **④** | yes | ANG | alive | image | 22 |
| 23 | 45 | male | STEMI | LCA/RCA | yes | **①** | yes | CT | alive | image | 23 |
| 24 | 53 | female | STEMI | RCA | yes | **④** | no | ANG | alive | image | 24 |
| 25 | 67 | female | STEMI | RCA | yes | **④** | no | CT | alive | image | 25 |
| 26 | 52 | female | STEMI | LCA | yes | **④** | yes | ECHO | alive | image | 26 |
| 27 | 54 | male | STEMI | LCA | yes | **①** | no | ANG and ECHO | dead | image | 27 |

| **Patient Number** | **Age** | **Gender** | **Type of AMI** | **Involved**  **coronary**  **artery** | **Cath-lab clues or not** | **Type of**  **clues** | **Stents were implanted or not** | **Method of diagnosis** | **Clinical outcome** | **Involved**  **media** | **Reference** |
| --- | --- | --- | --- | --- | --- | --- | --- | --- | --- | --- | --- |

| 28 | 69 | male | STEMI | RCA | yes | **④** | yes | IVUS | unknown | image and video | 28 |
| --- | --- | --- | --- | --- | --- | --- | --- | --- | --- | --- | --- |

**Abbreviations:** AMI, acute myocardial infarction; STEMI, ST-segment elevated myocardial infarction; NSTEMI, non-ST-segment elevated myocardial infarction; LCA, left coronary artery; RCA, right coronary artery; CT, computed tomography; ANG, angiogram; ECHO, echocardiogram; IVUS, intravenous ultrasound; Type of angiographic clues: ①Catheters cannot be advanced to the coronary ostia easily; ②Significant catheter swing within the aorta; ③Aortic angiography shows a non-smooth aortic wall; ④Coronary "thin strip of stenosis"; ⑤Pseudo-occlusion of the coronary ostium.

**Reference**

1. Adiarto S, Kurnianingsih N, Prasetya I, Nugroho FW, Uberoi R. Successful Primary PCI in Stanford Type A Aortic Dissection Complicated by Inferior ST-Elevation Myocardial Infarction: A Case in a Facility with No Surgical Backup. Int J Angiol 2024;33:62-65.

2. Yu J, Yu W, Zeng H, Gao J, Xiong J. Case report: Surgery combined with extracorporeal membrane oxygenation for a patient with type A aortic dissection complicated with myocardial infarction after percutaneous coronary intervention. Front Cardiovasc Med 2023;10:1205373.

3. Uehara H, Okuyama M, Oe Y, Yoshimura T, Gunji T. Acute aortic dissection with left coronary artery obstruction. Clin Case Rep 2023;11:e7719.

4. Fu D, Beccarino N, Ali I, Xu J, Pena I, Meraj P. A Case of Complex Percutaneous Coronary Intervention for ST Elevation Myocardial Infarction Caused by Extension of Type A Aortic Dissection into the Left Main Coronary Artery. Cardiovasc Revasc Med 2023;53s:S279-s282.

5. Chung K, Huang P, Lee K. Percutaneous Coronary Intervention as a Bridge Therapy for Aortic Dissection Related Bilateral Coronary Malperfusion: A Case Report. Heart Surg Forum 2023;26:E040-e042.

6. Zhang Q, Yang DD, Xu YF, Qiu YG, Zhang ZY. De Winter electrocardiogram pattern due to type A aortic dissection: a case report. BMC Cardiovasc Disord 2022;22:150.

7. Abdelwahed YS, Weber AC, Siegrist PT. Left Main Ischemia as First Manifestation of Aortic Dissection. JACC Cardiovasc Interv 2021;14:476-480.

8. Huang CY, Hung YP, Lin TH, Chang SL, Lee WL, Lai CH. Catheter directed diagnosis of ST-segment elevation myocardial infarction induced by type A aortic dissection: A case report. Medicine (Baltimore) 2020;99:e18796.

9. Chen HY, Lu DY, Sung SH. Acute myocardial infarction and coronary intramural haematoma: a clue to aortic dissection. EuroIntervention 2019;14:e1852-e1853.

10. Shibata Y, Kobayashi N, Hata N, Shimizu W. Optical coherence tomography-guided percutaneous coronary stenting for right coronary artery malperfusion due to acute type A aortic dissection. Coron Artery Dis 2018;29:605-606.

11. Fujiyoshi T, Koizumi N, Nishibe T, Sugiyama K, Ogino H. Direct Repair of Localized Aortic Dissection with Critical Malperfusion of the Left Main Trunk. Ann Thorac Cardiovasc Surg 2018;24:320-323.

12. Ibrahim M, Rashid J, Kurtz SC, Mungee S. The telescoping aorta: a case of circumferential aortic dissection with intimo-intimal intussusception into the left ventricle. BMJ Case Rep 2017;2017.

13. Takahashi K, Inaba S, Kikuchi K et al. Intravascular Ultrasound-Diagnosed Acute Aortic Dissection Involving Left Main Closure. JACC Cardiovasc Interv 2016;9:1631-2.

14. Chen A, Ren X. Aortic Dissection Manifesting as ST-Segment-Elevation Myocardial Infarction. Circulation 2015;131:e503-4.

15. Ahn JH, Park JR, Koh JS et al. Paraparesis after Primary Percutaneous Coronary Intervention for ST-segment Elevation Myocardial Infarction: Combined Uncommon Complications of Acute Aortic Syndrome in a Patient. Intern Med 2015;54:2191-5.

16. Wu BT, Li CY, Chen YT. Type A Aortic Dissection Presenting with Inferior ST-Elevation Myocardial Infarction. Acta Cardiol Sin 2014;30:248-52.

17. Kim SH, Kim KH, Lim SW, Kim IJ, Moon JY, Cha DH. IVUS images of the left main intramural hematoma from aortic dissection. Int J Cardiol 2014;173:e27-30.

18. Lentini S, Specchia L, Cricco A et al. Hybrid management of acute type A aortic dissection presenting as acute coronary syndrome. Int J Cardiol 2013;167:e85-7.

19. Okamoto M, Amano T, Matsuoka S et al. A Case of Acute Myocardial Infarction due to Left Main Trunk Occlusion Complicated With Aortic Dissection as Diagnosed by Intravascular Ultrasound. Cardiol Res 2012;3:232-235.

20. Lajevardi SS, Sian K, Ward M, Marshman D. Circumferential intimal tear in type A aortic dissection with intimo-intimal intussusception into left ventricle and left main coronary artery occlusion. J Thorac Cardiovasc Surg 2012;144:e21-3.

21. Cockburn JA, Munir SM, Prendergast BD. Novel use of a guiding catheter to relieve left main stem occlusion complicating Stamford type A aortic dissection. J Cardiol Cases 2012;6:e23-e25.

22. Saxena P, Boyle A, Shetty S, Edwards M. Left main coronary artery stenting prior to surgical repair of a type a aortic dissection. J Card Surg 2011;26:634-5.

23. Chamnarnphol N, Cheewatanakornkul S, Wisaratapong T. Coronary spasm due to type A aortic dissection complicated by hemopericardium: a case report of another possible cause of coronary malperfusion. Intern Med 2010;49:829-31.

24. Arrivi A, Tanzilli G, Tritapepe L, Mazzesi G, Mangieri E. Undetected acute aortic dissection in a patient referred for primary coronary angioplasty: a successful treatment of perioperative bleeding after abciximab administration. BMJ Case Rep 2010;2010.

25. Sir JJ, Kim YI, Cho WH, Choi SK. Acute myocardial infarction due to aortic dissection. Intern Med 2009;48:173.

26. Camaro C, Wouters NT, Gin MT, Bosker HA. Acute myocardial infarction with cardiogenic shock in a patient with acute aortic dissection. Am J Emerg Med 2009;27:899.e3-6.

27. Zegers ES, Gehlmann HR, Verheugt FW. Acute myocardial infarction due to an acute type A aortic dissection involving the left main coronary artery. Neth Heart J 2007;15:263-4.

28. Na SH, Youn TJ, Cho YS et al. Images in cardiovascular medicine. Acute myocardial infarction caused by extension of a proximal aortic dissection flap into the right coronary artery: an intracoronary ultrasound image. Circulation 2006;113:e669-71.
